# Genetic Counseling and Genetic Testing for Familial Hypercholesterolemia

**DOI:** 10.3390/genes15030297

**Published:** 2024-02-26

**Authors:** Hayato Tada, Masa-aki Kawashiri, Atsushi Nohara, Tomoko Sekiya, Atsushi Watanabe, Masayuki Takamura

**Affiliations:** 1 Division of Cardiovascular Medicine, Kanazawa University Graduate School of Medicine, Kanazawa 920-8640, Japan; masayuki.takamura@gmail.com; 2 Department of Internal Medicine, Kaga Medical Center, Kaga 922-8522, Japan; masaakikawashiri@gmail.com; 3 Department of Clinical Genetics, Ishikawa Prefectural Central Hospital, Kanazawa 920-8530, Japan; atsushinohara@mac.com; 4 Division of Clinical Genetics, Kanazawa University Hospital, Kanazawa 920-8641, Japan; ptomop.m.1984.02.15@gmail.com (T.S.); aw3703@staff.kanazawa-u.ac.jp (A.W.)

**Keywords:** familial hypercholesterolemia, LDL, genetics, genetic counseling, genetic testing

## Abstract

Familial hypercholesterolemia (FH) is one of the most common autosomal codominant Mendelian diseases. The major complications of FH include tendon and cutaneous xanthomas and coronary artery disease (CAD) associated with a substantial elevation of serum low-density lipoprotein levels (LDL). Genetic counseling and genetic testing for FH is useful for its diagnosis, risk stratification, and motivation for further LDL-lowering treatments. In this study, we summarize the epidemiology of FH based on numerous genetic studies, including its pathogenic variants, genotype–phenotype correlation, prognostic factors, screening, and usefulness of genetic counseling and genetic testing. Due to the variety of treatments available for this common Mendelian disease, genetic counseling and genetic testing for FH should be implemented in daily clinical practice.

## 1. Introduction

Familial hypercholesterolemia (FH) is one of the most common autosomal codominant forms of Mendelian disease. The major complications of FH include tendon and cutaneous xanthomas and coronary artery disease (CAD) associated with substantial elevation of serum low-density lipoprotein cholesterol [1,2]. There are four known causative genes, including the LDL receptor gene (*LDLR*), apolipoprotein B (*APOB*), proprotein convertase subtilisin/kexin type 9 (*PCSK9*), and LDL receptor adaptor protein 1 (*LDLRAP1*). Among them, LDLR is by far the most common cause, the malfunction of which leads to the disruption of LDL catabolism, thus elevating LDL-C levels [3]. Currently, the prevalence of this disease is estimated to be 1 in 300 among the general population based on a meta-analysis of genetic epidemiological studies [4]. Genetic testing for FH was introduced in research many years ago, and it can now be performed in a clinical setting. In addition, several different types of LDL-lowering therapies that can adequately reduce LDL cholesterol have been developed over the past decades, including statins, ezetimibe, resins, PCSK9 inhibitors, microsomal triglyceride transfer protein (MTTP) inhibitors, and angiopoietin-like protein 3 (ANGPTL3) inhibitors [5,6,7,8,9]. Now that we have tools for the genetic diagnosis of and effective treatments for this common genetic disease, we can summarize the current status of genetic counseling and genetic testing in FH, mainly based on our experience and on the experience of other collaborators across the world. We believe that it is now the best time to implement genetic counseling and genetic testing for FH in daily clinical practice.

## 2. Genetic Epidemiology and Cardiovascular Disease of FH

In the past, epidemiological studies without genetic status estimated the prevalence of FH to be 1 in 500 among the general population [10]. The clinical diagnostic criteria usually include elevated LDL cholesterol, family history, and tendon/cutaneous xanthomas [11,12,13]. Conversely, a genetic epidemiological study in the Hokuriku district of Japan revealed that the prevalence of heterozygous FH among the general population was 1 in 208 [14]. Similar results have been shown in case–control studies, hospital-based studies, and a universal screening [15,16,17]. In fact, a meta-analysis including more than 11 million individuals revealed that its prevalence based on genetic epidemiology is 1 in 300 among the general population [4]. This discrepancy can be attributed to the low sensitivity of clinical diagnostic criteria. In fact, despite being a highly specific physical finding in FH, the presence of tendon and/or cutaneous xanthomas reportedly varies from 5% to 60%, depending on age [18]. By contrast, genetic epidemiology was able to objectively reveal the prevalence of FH among the general population, even among individuals without any typical physical findings of FH. Given that genetic testing for FH has a sensitivity of 60–80%, the true prevalence of FH can be even higher. Aside from this, the major complications of FH should also be considered, specifically cardiovascular disease, due to its high mortality burden, which can be offset with early diagnosis and treatment [19]. Considering all these factors together, the prevalence of heterozygous FH and homozygous FH in the general population is currently estimated at 1 in 300 and at 1 in 360,000 across the world, except for some populations where founder effects are observed, including French Canadians, Afrikaners in South Africa, or Christian Lebanese [4].

## 3. Pattern of Inheritance and Pathogenic Variants of FH

FH has been recognized as an autosomal codominant inherited disease, except in extremely rare situations of individuals with pathogenic variants of LDLRAP1, wherein an autosomal recessive inheritance pattern is observed [20]. Nevertheless, we can identify relatives who are affected when we diagnose a patient with FH through cascade screening because the disease has a 100% penetration rate, and only a few cases with de novo pathogenic variants have been described [21]. Genetic testing is usually not conducted for the relatives of the proband of FH because the extremely high level of LDL cholesterol is sufficient to make a diagnosis.

When it comes to genetic testing, it is almost always very difficult to determine the pathogenicity of genetic variants for certain diseases [22]. There have been a substantial number of investigations and reports on the pathogenic variants of FH [23,24,25,26,27,28]. We have previously summarized pathogenic variants in LDLR among Japanese FH patients and cataloged 132 of these, including those specific to Japanese populations [20]. Because of this background, genetic counseling and genetic testing has been covered by the Japanese National Health Insurance since 2022.

## 4. Genotype–Phenotype Correlations between FH and Homozygous FH

Genotype–phenotype correlations exist in some diseases and genes [29], such as in FH. For example, the phenotype of FH caused by nonsense variants in the *LDLR* is much worse than that caused by missense variants [30]. Another association study revealed that the effect size for “FH-variant” coronary artery disease was inversely associated with their allele frequency [31], which is in accordance with the concept that rarer genetic variants tend to have larger effect sizes on diseases [32]. Therefore, we believe that each rare variant associated with FH has different effect sizes on LDL cholesterol and coronary artery disease according to their allele frequency as well as its effect on protein.

**Figure 1:**
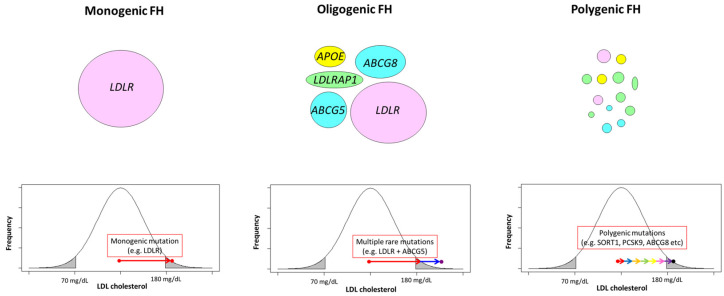
Monogenic, oligogenic, and polygenic FH. Monogenic FH is caused by a rare variant with a large effect size. Oligogenic FH is caused by a rare variant with a large effect size and rare variant/s with moderate effect sizes. Polygenic FH is caused by multiple common variants with small effect sizes.

In addition to the genotype–phenotype correlations of each variant, there are different types of FH according to the different patterns of the accumulation of genetic variants. The simplest form is true homozygous FH, wherein the same biallelic pathogenic variants cause homozygous FH. In addition, there are several different types of homozygous FH, including compound heterozygous FH, caused by different pathogenic variants in an FH gene, and double heterozygous FH, caused by different pathogenic variants in different genes (e.g., *LDLR* and *PCSK9*). Importantly, the most severe phenotype is seen in homozygous FH with true homozygous variants in *LDLR*, followed by compound heterozygous variants in *LDLR*, then by double heterozygous variants in *LDLR* and *PCSK9*, which is the least severe [33]. However, it is important to remember that the phenotype of homozygous FH, regardless of genotype, is generally severe, with as much as 70% of homozygous FH patients with a median age of 27 years developing CAD [34]. Thus, it is important to recognize if the patient specifically has homozygous FH. The phenotype of FH is also affected by other genes, including ATP-binding cassette subfamily G member 5 (*ABCG5*), ATP-binding cassette subfamily G member 8 (*ABCG8*), and apolipoprotein E (*APOE*), which are known to be causal genes of recessive types of inherited dyslipidemia (Figure 1) [35,36]. Such cases are termed oligogenic FH, wherein a pathogenic variant is FH, and pathogenic variants are recessive types of inherited dyslipidemia that affect the phenotype. Currently, oligogenic FH is not classified as homozygous FH, but their phenotypes are worse than that of simple heterozygous FH, suggesting the need to classify different types of inherited dyslipidemia.

## 5. Factors Associated with FH Prognosis

**Figure 2:**
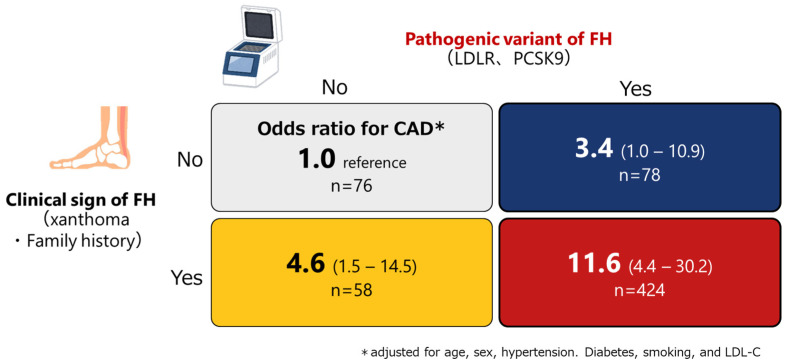
Impact of FH variants and clinical signs of FH on CAD. The 2 × 2 matrix represents the odds ratio for CAD wherein individuals without the FH variant and clinical signs of FH are used as a reference.

**Figure 3:**
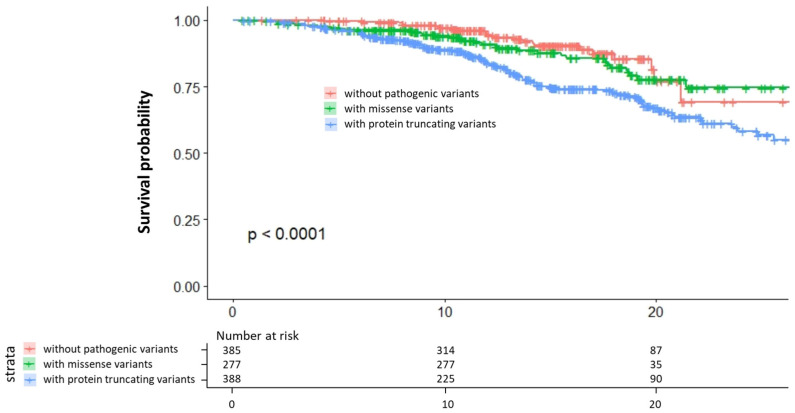
Prognosis of patients with FH according to genotype. Red: patients without pathogenic variants. Green: patients with missense variants. Blue: patients with protein-truncating variants.

The most important and prognosis-defining complication of FH is cardiovascular disease. The factors associated with CAD in patients with FH have long been investigated. Classic risk factors associated with CAD among the non-FH population, including age, male sex, smoking, hypertension, and diabetes, are significantly and independently associated with CAD among FH patients as well [37,38]. In addition, emerging risk factors, such as remnant cholesterol, HDL function, and lipoprotein (a) (Lp(a)), are also associated with CAD among patients with FH [39,40,41]. The International Atherosclerosis Society defines severe FH based on these factors, which are useful in identifying patients with an even higher risk for CAD [42]. Furthermore, the genetic status of FH has been associated with a 3- to 4-fold increased risk for CAD beyond the classical risk factors (Figure 2) [43]. In addition to the presence of FH variants, the type of pathogenic variants as well as other genetic situations, including polygenic risk scores comprising common genetic variations, are all associated with the phenotype [44]. We have shown that patients with FH caused by a protein-truncating variant had poorer prognosis than patients with FH caused by a missense variant (Figure 3) [30]. Accordingly, genetic backgrounds need to be considered during risk assessment in patients with FH [45].

## 6. Cascade Screening and Universal Screening for FH

Typically, patients with heterozygous FH start to develop myocardial infarction at the age of 30 and 50 years in men and women, respectively [46]. Moreover, we have shown that coronary atherosclerosis starts to develop at the age of 23 and 34 years in males and females, respectively [47]. Making an early diagnosis and initiating LDL-lowering therapy are key to improving the prognosis of patients with FH. Clinical guidelines worldwide are now recommending that treatment start at 8–10 years old [48]. In fact, we have shown that patients with heterozygous FH who started treatment before the age of 20 had a much better prognosis versus those who started treatment afterward [19]. Therefore, it is important to screen patients with FH in real-world settings, given that an early diagnosis leads to a better prognosis and that this is one of the most common inherited diseases. Currently, there are two major ways to screen for FH, cascade screening and universal screening.

Cascade screening involves screening the asymptomatic relatives of probands who seek medical attention; given its inheritance pattern and 100% penetrance rate, relatives have 50% chance of having FH. However, genetic testing is not always needed in cascade screening because high LDL cholesterol levels and a positive family history are sufficient to clinch the diagnosis. In our experience, patients with heterozygous FH who were diagnosed via cascade screening exhibited a much better prognosis than the probands [49].

Universal screening is another way to identify patients with FH very early in the clinical course. This involves testing LDL cholesterol levels in individuals at certain ages; then, those above a certain threshold are examined for FH [50]. There are several different strategies worldwide in terms of target age, target people, and LDL cholesterol threshold that are examined in detail. We have shown that universal screening for FH at the age of 9 or 10 (i.e., at the elementary school age) with an LDL cholesterol threshold of 140 mg/dL plus an option of genetic testing was useful for identifying pediatric FH patients and adult FH patients, their parents, via “reverse cascade screening”. By adopting reverse cascade screening, this strategy appears to be quite cost-effective, even accounting for the cost of genetic testing in Japan [51]. Alternatively, physical xanthomas, including Achilles tendon thickness, are not a reliable way to clinch the diagnosis in pediatric FH patients because these are only overt in adulthood [52,53,54,55,56,57,58,59].

## 7. The Impact of Genetic Counseling and Genetic Testing on LDL Cholesterol and Satisfaction

**Figure 4:**
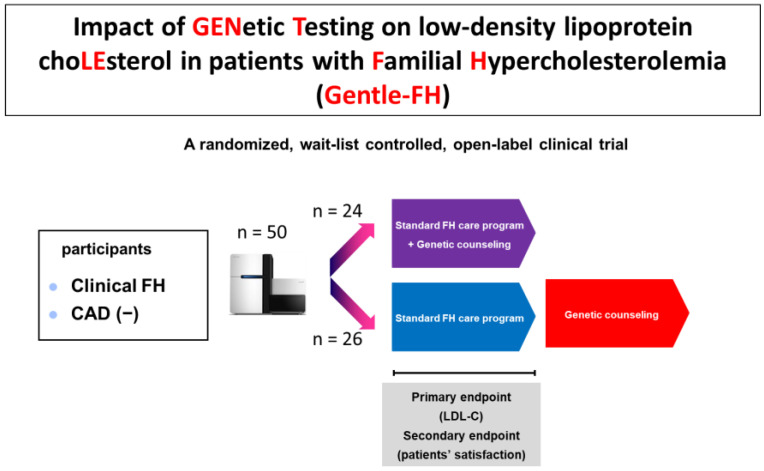
Schematic of the Gentle-FH trial.

**Figure 5:**
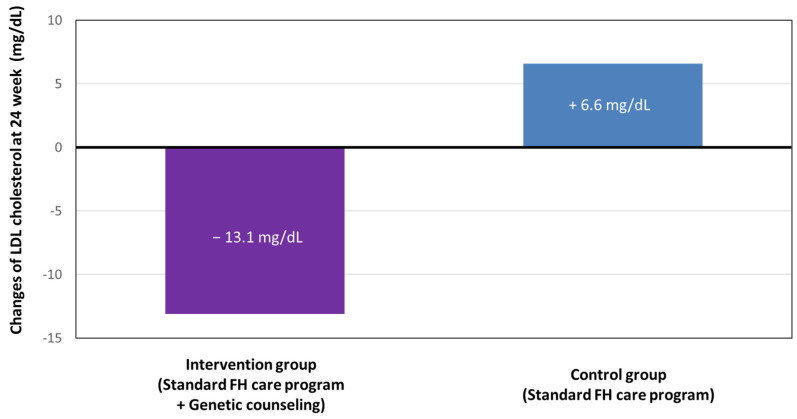
Primary endpoint (changes in LDL cholesterol) of the Gentle-FH trial. Purple: intervention group. Blue: control group.

As stated above, the utility of genetic counseling and genetic testing for FH are very clear. To confirm its usefulness, we conducted a randomized, waitlist-controlled, open-label clinical trial (Impact of GENetic Testing on low-density lipoprotein choLEsterol in patients with Familial Hypercholesterolemia [Gentle-FH: jRCTs04218002]) to determine the efficacy of providing genetic counseling on future cardiovascular risk based on genetic testing, in addition to a standard FH education program (Figure 4) [60]. In the intervention group, we reported a future cardiovascular risk based on genetic testing, which was added to the standard FH education at week 0. The waitlist control group was only given standard FH education according to the guidelines at week 0; they later underwent a genetic testing-based cardiovascular risk assessment at week 24. The primary endpoint of this study was the plasma LDL cholesterol level at week 24. We found that the intervention group had a significantly greater reduction in LDL cholesterol levels than the waitlist control group (mean changes, −13.1 vs. 6.6 mg/dL; difference, −19.7 mg/dL; 95% confidence interval, −34 to −5.6; *p* = 0.009; Figure 5) without affecting patient satisfaction [61]. Thus, we now have clinical evidence that providing genetic counseling, and genetic testing is useful for controlling LDL cholesterol, the most important surrogate marker of FH.

## 8. Sitosterolemia as a Phenocopy of FH

It is important to note that there is an important “phenocopy” of FH called sitosterolemia. Sitosterolemia is considered an extremely rare recessive disorder caused by pathogenic variants in *ABCG5* or *ABCG8* in both alleles [62]. As mentioned above, a deleterious variant in ABCG5 or ABCG8 affects the phenotype of FH by further increasing LDL cholesterol [35]. When a patient exhibits pathogenic variants in both alleles of *ABCG5* or *ABCG8*, his or her LDL cholesterol level then typically increases to the level of homozygous FH. It has been shown that patients with sitosterolemia typically exhibit severely elevated LDL cholesterol, tendon xanthomas, and premature CAD, which are quite similar to homozygous FH [62]. In general, it is difficult to differentiate sitosterolemia from homozygous FH only by the clinical presentations stated above, because measuring serum sitosterol is uncommon in clinical practice, and it is sometimes very difficult to collect family history information. Accordingly, genetic testing for such patients, especially those suspected to have homozygous FH, is quite useful in terms of their differential diagnosis of sitosterolemia and homozygous FH. Currently, sitosterolemia is considered to be quite rare; however, its actual prevalence appears to be much higher based on an estimation using a genome database. It is estimated that at least 1 in 200,000 people in the general population have sitosterolemia caused by pathogenic variants in *ABCG5* or *ABCG8* [62]. Therefore, patients with sitosterolemia are likely to be misdiagnosed as having FH or simply misdiagnosed as having general hypercholesterolemia. To diagnose patients with sitosterolemia is quite important because there is a useful drug (ezetimibe) for them. In fact, LDL cholesterol levels in patients with sitosterolemia can be reduced dramatically either by diet or use of ezetimibe because of the fact that the cause of their hyper LDL cholesterolemia is the malfunction of the secretion of LDL cholesterol, and, thus, LDL cholesterol levels can be normalized if the intake of LDL cholesterol is strictly regulated.

To estimate the presence of deleterious *ABCG5* or *ABCG8* variant(s), it is useful to measure serum sitosterol levels. We have shown that a serum sitosterol level of 5 μg/mL or more is a useful “marker” for the presence of deleterious *ABCG5* or *ABCG8* variant(s) [63]. In addition, we can assume that patients whose serum sitosterol level is 10 μg/mL or more are highly likely to have sitosterolemia and have deleterious *ABCG5* or *ABCG8* variants in both alleles.

In addition to the information above, we and several others have provided useful information regarding the phenotype and genotype of sitosterolemia [64]. It has been shown that sitosterolemia exhibits blood abnormalities and arthritis in addition to xanthomas and premature CAD. Among the pathogenic variants, particular missense mutation (c.1166G > A/p.Arg389His) was the most frequently observed. This mutation has been found in sitosterolemia in other East Asian countries, including China and the Republic of Korea [64].

## 9. Lysosomal Acid Lipase Deficiency (LAL-D) as a Phenocopy of FH

Lysosomal acid lipase deficiency (LAL-D) is a rare, autosomal recessive disease involving lysosomal accumulation of cholesteryl esters and triglycerides [65]. Pathogenic variants in the lipase A, lysosomal acid type (*LIPA*) lead to a marked decrease in or the total absence of lysosomal acid lipase (LAL) activity, leading to lysosomal accumulation of cholesteryl esters and triglycerides. There are two types in LAL-D, including Wolman disease, which typically presents in the first month of life as a rapidly progressive disease with hepatosplenomegaly, malabsorption, and growth failure, with it being generally fatal in the first year of life [65]; and childhood/adult-onset LAL-D, formerly known as cholesteryl ester storage disease (CESD), which has a more variable course but commonly includes hepatosplenomegaly, chronic liver injury, elevated LDL cholesterol levels and triglycerides, and low HDL cholesterol levels [66]. There are cases where FH was suspected, which ultimately became diagnosed as LAL-D [66]. It is important to note that now we have a specific treatment called Sebelipase alfa, a recombinant human lysosomal acid lipase. Studies have shown an improvement in lipid parameters and liver enzymes [67,68].

## 10. Determination of Pathogenic Variants of FH

It is both important and difficult to determine the pathogenic variants of a certain inherited disorder. ACMG guidelines are typically used to clarify genetic variants, and these variants are typically classified into pathogenic variants of uncertain significance (VUS) and benign variants. To determine the pathogenicity of genetic variants, a variety of ways are considered, including the allele frequency of a general population, in silico predictive algorithms, types of variants, co-segregation with the disease in multiple affected family members, in vitro or in vivo functional studies, and a pathogenic variant database of certain diseases. In the case of FH, there are many reliable reports of pathogenic variants, especially in *LDLR* because of the abundance of clinical and genetic data. In fact, we have summarized the pathogenic variants in *LDLR* among Japanese FH patients and catalogued 132 pathogenic variants in *LDLR* [18]. Interestingly, a certain nonsense variant in *LDLR* (p.Lys811Ter) is quite frequently observed in a certain area of Japan (Hokuriku area), probably due to the founder effect, whereas many singleton variants were identified in Kansai, reflecting the dynamic population migrations from all over Japan. [18]. This type of database is useful when medical geneticists try to determine the pathogenicity of genetic variants. On the other hand, it is still difficult to determine the pathogenicity of genetic variants in *PCSK9* or *APOB* due to lack of clinical as well as genetic data. In the case of *LDLR*, it is rather easy to determine the pathogenicity of the genetic variants since a loss of function leads to the disease. However, in the case of *PCSK9* and *APOB*, a loss of function leads to the opposite phenotype, namely, hypobetalipoproteinemia [69]. In fact, PCSK9 works to help degrade LDLR, and, thus, a loss of function of *PCSK9* leads to an increase in LDLR. On the other hand, gain-of-function variants in *PCSK9* lead to FH. However, it is very difficult to estimate the function of PCSK9 because of the complicated mechanisms of PCSK9 in vivo. In the case of *APOB*, a certain variant (p.Arg3527Gln) has been shown to be a pathogenic variant as FH because of the disruption in the interaction between APOB particles and LDLR [1]. However, it is very difficult to ascertain the pathogenicity of the other genetic variants in APOB for FH. Moreover, *APOB* is a very large gene, and, thus, there are at least some genetic variants in any individual. This situation sometimes causes very confusing situations where apparently benign variants are reported as pathogenic variants of FH. We need to keep in mind that classification of pathogenic variants of FH is rather easy in *LDLR*, whereas it is difficult in *PCSK9* and *APOB*.

## 11. Medical Therapies for FH

As stated above, FH should be diagnosed as early as possible, and should be treated according to the guidelines. We presently have a variety of medications to reduce LDL cholesterol in patients with FH, including statins, ezetimibe, resin, probucol, PCSK9 inhibitors, lomitapide (MTTP inhibitor), and evinacumab (ANGPTL3 inhibitor). When these medications are not good enough, lipoprotein apheresis should be then introduced. Statins are the primary medication for patients with FH and other types of hyper LDL cholesterolemia. It has been shown that the effectiveness of statins for heterozygous FH to reduce LDL cholesterol is as effective as for general hyper LDL cholesterolemia. Therefore, the main questions are (1) when to start to treat them, (2) what the LDL cholesterol treatment target is, and (3) what the next steps after the introduction of statins are. Regarding the timing of the introduction of medical therapies for heterozygous FH, current guidelines now recommend starting treatments at 8 to 10 years of age [13]. This is based on several observations, including an observation that earlier treatment led to much better prognosis; statins have been shown to be quite safe for pediatric FH patients, and carotid and coronary plaque appear to start to develop at 20 years of age in patients with heterozygous FH. However, it is important to keep in mind that statins are now currently contraindicated in pregnant individuals in many countries, even as there is no apparent clinical evidence suggesting the risk of statins in this situation [70]. In addition, we need to be careful to use statins for patients with homozygous FH since statins are not as effective as other situations because the effect of this drug is dependent on residual LDLR activity. Therefore, statins may have no effect at all for the patients with homozygous FH whose LDLR activity is null [33]. We can estimate the effectiveness of statins for homozygous FH based on the results of genetic testing. As for ezetimibe, it has also been shown to be quite useful to reduce LDL cholesterol in patients with FH as well. When the effectiveness of ezetimibe is unexpectedly high, we then need to consider a diagnosis of sitosterolemia because of the reasons stated above. Resin is a drug long since used to reduce LDL cholesterol levels by 15 up to 20%. It is of note that the effect of this drug is independent of statins and ezetimibe; therefore, a combination therapy of statins, ezetimibe, and resin can additively reduce LDL cholesterol. We have shown that this combination therapy can reduce the LDL cholesterol level of heterozygous FH by 66% [71]. Probucol is a potent antioxidant and reduces tendon xanthomas in FH patients despite the reduction in HDL cholesterol levels. PCSK9 inhibitors have also been quite effective in reducing LDL cholesterol level in patients with heterozygous FH [72]. In fact, most of the patients with heterozygous FH who attained their LDL cholesterol treatment under secondary prevention settings and whose LDL cholesterol treatment target was below 70 mg/dL actually used PCSK9 inhibitors [73]. Therefore, PCSK9 inhibitors should be introduced for patients with FH—especially those under secondary prevention settings. Moreover, there are several ways of inhibiting PCSK9, including via monoclonal antibodies and siRNA. There are a couple of other options targeting PCSK9 that will be available in the near future. However, we need to keep in mind that the effect of PCSK9 inhibitors also depends on their residual LDLR activity, and, thus, its effectiveness is quite limited in cases of homozygous FH patients, especially those whose residual LDLR activity is null [1]. Conversely, when we find a patient who exhibits resistance to PCSK9 inhibitors, genetic testing for FH should then be considered. In addition, it is quite important to note that patients with homozygous FH should be treated as soon as they are diagnosed regardless of their age. There are a couple of options for patients with homozygous FH whose residual LDLR activity is null. The first option is lomitapide (MTTP inhibitor). Lomitapide is a drug targeting MTTP, which plays a key role in the synthesis of very-low density lipoprotein (VLDL) in the liver and chylomicron in the intestine [8]. Therefore, the effect of this drug is independent of the LDLR pathway. It has been shown that ~50% reduction can be obtained when using this drug in homozygous FH patients [8]. Although this use of this drug has been sometimes associated with appetite loss and diarrhea, gradual dose titration of this drug appears to be quite useful in preventing these side effects [8]. The second option is evinacumab (ANGPTL3 inhibitor). Recently, ANGPTL3 has emerged as a target for the treatment of elevated LDL cholesterol levels based on the interesting findings of familial combined hypolipidemia with biallelic loss-of-function pathogenic variants in the *ANGPTL3* who exhibited extremely low LDL cholesterol levels and a substantially reduced risk of atherosclerotic cardiovascular disease [74]. The inhibition of ANGPTL3 reduces LDL cholesterol independent of the LDLR pathway, possibly by promoting the removal of VLDL before it can be converted to LDL. Evinacumab is a fully human monoclonal antibody that selectively binds to and inhibits ANGPTL. It has been shown that this drug can effectively reduce LDL cholesterol by ~50%, even in the patients with homozygous FH whose residual LDLR activity is null [5]. In addition, this drug has safely and effectively reduced LDL cholesterol in pediatric homozygous FH patients aged 5 to 11 years [75]. Another option is lipoprotein apheresis. Although this is an invasive treatment, it can not only reduce LDL cholesterol but also other atherogenic lipoproteins, such as Lp(a), substantially. The clinical usefulness of this procedure has been already shown many times due to the fact that lipoprotein apheresis improves blood rheology, reduces oxidative stress parameters, and improves endothelial function [76].

## 12. LDL Cholesterol Target Attainment in FH

Given that LDL cholesterol is the primary causal factor of atherosclerosis in patients with FH, clinical guidelines across the world clearly stipulate their LDL cholesterol treatment target. Despite the importance of LDL cholesterol-lowering in patients with FH, it has been shown that LDL cholesterol target attainment is quite low (2.7~31.9%) [73,77]. Although statin, ezetimibe, and colestimide are quite effective in reducing LDL cholesterol (up to 66%) [71], LDL cholesterol levels are typically too high in patients with FH to attain their LDL cholesterol treatment target. Therefore, newer drugs, including PCSK9 inhibitors and others documented above, are recommended if needed.

## 13. Conclusions

In 2014, the Japanese government decided to introduce a law on intractable diseases in Japan to cover the medical costs of intractable diseases, many of which are rare inherited diseases, including homozygous FH, sitosterolemia, and LAL-D. This law also stipulates that information regarding the clinical and genetic features of such intractable diseases should be continuously collected to improve the diagnosis of and therapies for these diseases. We now collect phenotypes and genotypes of this unique disease in a notion-wide manner in Japan [57]. Through this program, we have shown that (1) the median age of diagnosis is currently 27 years old, (2) 70% of the patients suffer from coronary artery disease, and (3) LDL cholesterol control is inadequate in patients with homozygous FH in Japan [34]. These data are quite important to understand the current situation of such rare disorders, and the Japanese government is currently trying to improve this situation with the Committee on Primary Dyslipidemia of the Ministry of Health, Labor, and Welfare of Japan under the Research Program on Rare and Intractable Disease.

Given that we have a variety of treatments for this common Mendelian disease, even for patients with homozygous FH, now is the prime time to implement genetic counseling and genetic testing for FH in daily clinical practice.

## Data Availability

Not applicable.
